# Whole-Genome Sequence of Phage-Resistant Strain Escherichia coli DH5α

**DOI:** 10.1128/genomeA.00097-18

**Published:** 2018-03-08

**Authors:** Jingchao Chen, Yi Li, Kun Zhang, Hailei Wang

**Affiliations:** a Singapore-China Joint Engineering Laboratory for Bioconversion Technology of Functional Microbes, College of Life Sciences, Henan Normal University, Xinxiang, China; b Advanced Environmental Biotechnology Center, Nanyang Environment and Water Research Institute, Nanyang Technological University, Singapore, Singapore

## Abstract

The genomes of many strains of Escherichia coli have been sequenced, as this organism is a classic model bacterium. Here, we report the genome sequence of Escherichia coli DH5α, which is resistant to a T4 bacteriophage (CCTCC AB 2015375), while its other homologous E. coli strains, such as E. coli BL21, DH10B, and MG1655, are not resistant to phage invasions. Thus, understanding of the genome of the DH5α strain, along with comparative analysis of its genome sequence along with other sequences of E. coli strains, may help to reveal the bacteriophage resistance mechanism of E. coli.

## GENOME ANNOUNCEMENT

Escherichia coli, generally known as E. coli, is the most common Gram-negative bacterium in warm-blooded animals (1). E. coli is recognized as one of the classic model creatures and is extensively used in various fields of the biological sciences (2, 3). E. coli is only one species of Enterobacteriaceae, although the physiological status of strains from different sources may vary dramatically. DH5α is a typical engineered E. coli widely used in the laboratory, since it allows exogenous plasmid DNA to be amplified inside its body. More specifically, a strain of DH5α preserved in our laboratory has resistance to a T4 phage (CCTCC AB 2015375). However, other E. coli strains such as BL21, DH10B, and MG1655, which are homologous to DH5α, do not resist the invasion of that phage.

The single-molecule PacBio sequencing technique, belonging to the third-generation sequencing technology, was adopted to measure the complete genome map of the DH5α strain. During sequencing, the Hierarchical Genome Assembly Process (HGAP) software was used to assemble the bacterial genome. After the sequencing, a total of 78,372 sequenced reads with an average length of 6,374.2 bp were obtained, and the sequencing depth reached 103.36×. The remaining contig formed a framework sequence (scaffold) without gaps. The sequenced genes were predicted with the software Glimmer 3.02 (http://ccb.jhu.edu/software/glimmer/index.shtml). BLAST 2.2.28+ was applied to predict protein sequences in the NCBI nr, KEGG, STRING, and GO databases for BLAST comparative analysis to obtain the predicted gene annotation information. Gene analyses related to phage resistance, such as prophage and clustered regularly interspaced short palindromic repeat (CRISPR) sequences, were analyzed by PHAST (http://phast.wishartlab.com/index.html) and CRISPRFinder (http://crispr.i2bc.paris-saclay.fr/) respectively (4, 5). The software programs tRNAscan-SE v1.3.1 and Barrnap 0.4.2 were used for tRNA and rRNA determination (6, 7).

The size of the total genome is 4,833,062 bp, and the genome contains a circular chromosome but is without a plasmid. The 4,833,062 bp of the chromosome has 4,636 genes, including 22 rRNA genes and 89 tRNA genes with an average length of 919.23 bp. The GC content of the chromosome is 50.752% and is nearly identical to the GC content of other E. coli strains that can be found in NCBI. Moreover, a total of 9 CRISPR sequences were found in the genes. Two of them are identified CRISPR sequences, and the remaining are questionable CRISPR sequences. In the whole genome, 5 prophage sequences were found, of which 4 are intact prophage sequences and the remaining 1 is an incomplete sequence. These prophage sequences correspond to the following phages: PHAGE_Entero_Sf101_NC_027398, PHAGE_Escher_pro483_NC_028943, PHAGE_Entero_lambda_NC_001416, PHAGE_Shigel_POCJ13_NC_025434, and PHAGE_Salmon_Fels_2_NC_010463.

These CRISPR sequences, as well as the prophage sequences, may be involved in bacterial resistance to phage (8, 9). The full-genome sequence of the DH5α strain may be employed to find phage resistance-related genes and determine the mechanisms at the molecular level of bacterial resistance to phages. Furthermore, the genome sequence information may also be utilized to evaluate the genetic diversity of various E. coli strains.

### Accession number(s).

The genome sequence of E. coli DH5α is available in GenBank under accession number CP026085.

## References

[1] Singleton P 1999 Bacteria in biology, biotechnology, and medicine 5th ed. Chichester, New York: John Wiley.

[2] Chaudhuri RR, Henderson IR 2012 The evolution of the *Escherichia coli* phylogeny. Infect Genet Evol 12:214–226. doi:10.1016/j.meegid.2012.01.005. 22266241

[3] Lee SY 1996 High cell-density culture of *Escherichia coli*. Trends Biotechnol 14:98–105. doi:10.1016/0167-7799(96)80930-9. 8867291

[4] Zhou Y, Liang Y, Lynch KH, Dennis JJ, Wishart DS 2011 PHAST: a fast phage search tool. Nucleic Acids Res 39:W347–W352. doi:10.1093/nar/gkr485. 21672955 PMC3125810

[5] Grissa I, Vergnaud G, Pourcel C 2007 CRISPRFinder: a web tool to identify clustered regularly interspaced short palindromic repeats. Nucleic Acids Res 35:W52–W57. doi:10.1093/nar/gkm360. 17537822 PMC1933234

[6] Schattner P, Brooks AN, Lowe TM 2005 The tRNAscan-SE, snoscan and snoGPS web servers for the detection of tRNAs and snoRNAs. Nucleic Acids Res 33:W686–W689. doi:10.1093/nar/gki366. 15980563 PMC1160127

[7] Lagesen K, Hallin P, Rødland EA, Stærfeldt H-H, Rognes T, Ussery DW 2007 RNAmmer: consistent and rapid annotation of ribosomal RNA genes. Nucleic Acids Res 35:3100–3103. doi:10.1093/nar/gkm160. 17452365 PMC1888812

[8] Horvath P, Barrangou R 2010 CRISPR/Cas, the immune system of Bacteria and Archaea. Science 327:167–170. doi:10.1126/science.1179555. 20056882

[9] Ali Y, Koberg S, Heßner S, Sun X, Rabe B, Back A, Neve H, Heller KJ 2014 Temperate *Streptococcus thermophilus* phages expressing superinfection exclusion proteins of the Ltp type. Front Microbiol 5:98. doi:10.3389/fmicb.2014.00098. 24659988 PMC3952083

